# Comparative Study of the Foaming Behavior of Ethylene–Vinyl Acetate Copolymer Foams Fabricated Using Chemical and Physical Foaming Processes

**DOI:** 10.3390/ma17153719

**Published:** 2024-07-27

**Authors:** Yaozong Li, Junjie Jiang, Hanyi Huang, Zelin Wang, Liang Wang, Bichi Chen, Wentao Zhai

**Affiliations:** School of Materials Science and Engineering, Sun Yat-sen University, Guangzhou 510275, China; liyz33@mail2.sysu.edu.cn (Y.L.); huanghanyi@sysunc.com (H.H.); wangzlin7@mail2.sysu.edu.cn (Z.W.); wangliang27@mail2.sysu.edu.cn (L.W.); chenbch23@mail2.sysu.edu.cn (B.C.)

**Keywords:** ethylene–vinyl acetate copolymer, physical foaming, crosslinking network, cell structure

## Abstract

Ethylene–vinyl acetate copolymer (EVA), a crucial elastomeric resin, finds extensive application in the footwear industry. Conventional chemical foaming agents, including azodicarbonamide and 4,4′-oxybis(benzenesulfonyl hydrazide), have been identified as environmentally problematic. Hence, this study explores the potential of physical foaming of EVA using supercritical nitrogen as a sustainable alternative, garnering considerable interest in both academia and industry. The EVA formulations and processing parameters were optimized and EVA foams with densities between 0.15 and 0.25 g/cm^3^ were produced. Key findings demonstrate that physical foaming not only reduces environmental impact but also enhances product quality by a uniform cell structure with small cell size (50–100 μm), a wide foaming temperature window (120–180 °C), and lower energy consumption. The research further elucidates the mechanisms of cell nucleation and growth within the crosslinked EVA network, highlighting the critical role of blowing agent dispersion and localized crosslinking around nucleated cells in defining the foam’s cellular morphology. These findings offer valuable insights for producing EVA foams with a more controllable cellular structure, utilizing physical foaming techniques.

## 1. Introduction

Ethylene–vinyl acetate copolymer (EVA) is an important class of thermoplastic elastomer (TPE) synthesized through the copolymerization of ethylene and vinyl acetate [1]. EVA combines the mechanical strength of ethylene units with the exceptional flexibility imparted by vinyl acetate units. EVA foams are extensively used in sports footwear and other sectors due to their low density, soft touch, and good cushioning properties. Traditionally, the production of EVA foams has been dominated by chemical foaming methods, supported by well-established technological systems. EVA foams prepared by chemical foaming have a density of about 0.15 to 0.25 g/cm^3^ and a ball rebound value of approximately 40% to 50% [2,3]. Over the past decade, with increasing environmental awareness and the rise of green manufacturing technologies, physical foaming has emerged as a promising technique [4]. Utilizing environmentally friendly supercritical fluids such as CO_2_, physical foaming facilitates the control of nucleation and growth processes in foam production, achieving a low density of 0.1 to 0.2 g/cm^3^ and an outstanding energy returned coefficient of 60% to 80% in TPE foams like thermoplastic polyurethane (TPU) [5,6], poly(ether-block-amide) (PEBA) [7], and thermoplastic poly(ether-ester) elastomer [8]. These novel foam materials are gradually challenging and partially replacing traditional EVA foams in the road racing shoe market [9,10].

Chemical blowing agents such as azodicarbonamide (AC) and 4,4′-oxybis(benzenesulfonyl hydrazide) are widely utilized in the preparation of EVA foams [11,12]. During heating, these agents decompose to produce nitrogen or other gasses, thereby forming cellular structures within the EVA matrix. However, the decomposition of chemical blowing agents may release harmful substances that pose potential threats to the environment and human health. Furthermore, due to the challenges in precisely controlling the chemical foaming process, the resulting EVA foam often exhibits uneven cellular structures, which adversely affect the foam’s resilience and mechanical properties [13,14]. To achieve sufficient strength and stable cellular structures, crosslinking agents and vulcanization activators are also necessary during the sample preparation process [15]. The extensive literature leverages blending and nanocomposite techniques to enhance the performance of EVA foams. Ma et al. [3] utilized TPU-grafted EVA (EVA-g-TPU) as a compatibilizer to improve the compatibility of EVA/TPU blended foams. As a result, the modified EVA/EVA-g-TPU/TPU foams exhibited enhanced mechanical properties, including increases in tensile strength, elongation at break, tear strength, and compression set by 19.0%, 9.3%, 43.6%, and 7.5%, respectively, without compromising other vital physical properties like density. PEBA [16] and polyolefin [17,18] are commonly blended with EVA to improve the physical properties of EVA foams. Lipik et al. [18] successfully reduced foam density while maintaining good dynamic energy absorption and energy return by blending a small amount of polyolefin elastomer (10–20 wt%) with EVA. Additionally, blending with a terpolymer elastomer resulted in approximately 16% increased compression strength, 27% increased hardness, and 9% enhanced dynamic energy return. Additionally, nanocomposite technology has also been proven to be an effective method for improving EVA foams. Kim et al. [13] found that the integration of multiwalled carbon nanotubes (MWCNT) into EVA foams significantly enhanced the mechanical resilience of the EVA/MWCNT foams. Notably, at a MWCNT loading of 5 phr, the compression set of EVA/MWCNT foams exhibited a substantial improvement of 30% over that of pure EVA foams. Similarly, many studies have found that various minerals and carbon materials play a positive role in enhancing the performance of EVA foams [2,19,20,21,22]. These studies have demonstrated that material modifications can enhance the performance of EVA foams produced through chemical foaming.

Compared to the chemical method, physical foaming often employs CO_2_ or N_2_ as blowing agents, which offer advantages such as eco-friendliness and precise control over the cellular structure. By meticulously regulating the foaming conditions, it is possible to optimize the cell structure, thereby enhancing the performance of EVA foams. Previous efforts by Zhang et al. [23] have shown that CO_2_ can be used as a blowing agent to produce EVA foams, exhibiting a resilience value of 48%. In contrast, supercritical N_2_ has shown a higher nucleation ability in the foaming of elastomeric materials [24,25]. Meanwhile, gas escape from elastomer foams prepared with N_2_ as the blowing agent is slower than with CO_2_ [24,26]. Sitz et al. [27] found that the cell structures of ethylene−propylene−diene monomer (EPDM) profile extrudates exhibited greater homogeneity when supercritical N_2_ was selected as the blowing agent compared to supercritical CO_2_. Even with a lower amount of supercritical N_2_, the extruded foam exhibited smaller-sized cells and higher cell density. Zhang et al. [28] developed EVA/polyolefin elastomer/styrene ethylene butylene styrene (SEBS) foams utilizing supercritical N_2_ foaming technology. Their findings showed that as the SEBS concentration increased, the uniformity of the cell size within the foam improved, and its density decreased significantly. Specifically, when the SEBS concentration reached 20 phr, the foam’s density was reduced from 0.19 to 0.13 g/cm^3^. This reduction in density is notably significant for enhancing the foam’s lightweight properties. Further research [29] revealed that due to the heterogeneous nucleation of hard segments in SEBS, the cell size of EVA/SEBS foams with a microcellular structure decreased and became more uniform as the SEBS content increased. At a SEBS concentration of 50 phr, the density of the blended foam was recorded at 0.17 g/cm^3^, demonstrating a further potential for density reduction and structural uniformity in high-SEBS-content foams.

The performance of foams is closely related to the properties of the materials themselves and the structure of the cells [30,31]. Wang et al. [32], for instance, have demonstrated that TPU foams with a gradient cellular structure increase the compression strength by 71.4% and modulus by 113.8%. For foams with the same expansion ratio, reducing cell size leads to enhanced compression strength, but deteriorated compression resilience [33]. Additionally, Huang et al. found that the physical properties of olefin block copolymers foams would also be affected by various bimodal cellular structures [34]. Although there has been significant progress in physical foaming technology for other TPE foams, research on the physical foaming behavior of EVA foam and its comparison with chemical foaming behavior remains relatively limited [35]. Furthermore, the decomposition process of chemical foaming agents and the selection of physical foaming agents have important effects on the final properties of EVA foams, but the specific mechanisms of these effects are not yet clear.

In this study, EVA samples with the same crosslinking content were used to fabricate various EVA foams using both chemical and physical foaming methods. Firstly, the effects of different crosslinking agent contents and crosslinking conditions on the vulcanization kinetics of EVA were characterized. Subsequently, a series of foaming experiments were conducted under different foaming conditions to prepare various EVA foams, and their cell morphology was evaluated. Finally, the nucleation and growth processes of cells during different foaming processes were further analyzed. By comparing the two foaming processes, this study aims to offer new insights and strategies for the green manufacturing and performance improvement of EVA foams.

## 2. Experimental

### 2.1. Materials

EVA raw resin (EVA 2518CO) with a VA content of 18% and a melt flow index of 2.5 g/10 min was obtained from Sipchem in the form of pellets. Bis(1-(tert-butylperoxy)-1-methylethyl)-benzene (BIPB), used as the crosslinking agent, was purchased from Xinnuo Qingdao Chemical Co., Ltd., Qingdao, China. Nano-sized titanium dioxide (TiO_2_) was purchased from US Research Nanomaterials, Inc., Houston, TX, USA. Calcium carbonate (CaCO_3_) with 98% purity was supplied by South Calcium Carbonate Co., Ltd., China. The industrial grade azodicarbonamide (AC) blowing agent, along with all other foaming auxiliaries, namely zinc oxide (ZnO), stearic acid (SA), and talc, were purchased from ANR Technologies Pte Ltd., Singapore. N_2_ with a purity of 99.9% was purchased from Guangzhou Guangqi Gas Corporation, Guangzhou, China.

### 2.2. Chemical and Physical Foaming of EVA Foams

**Figure 1:**
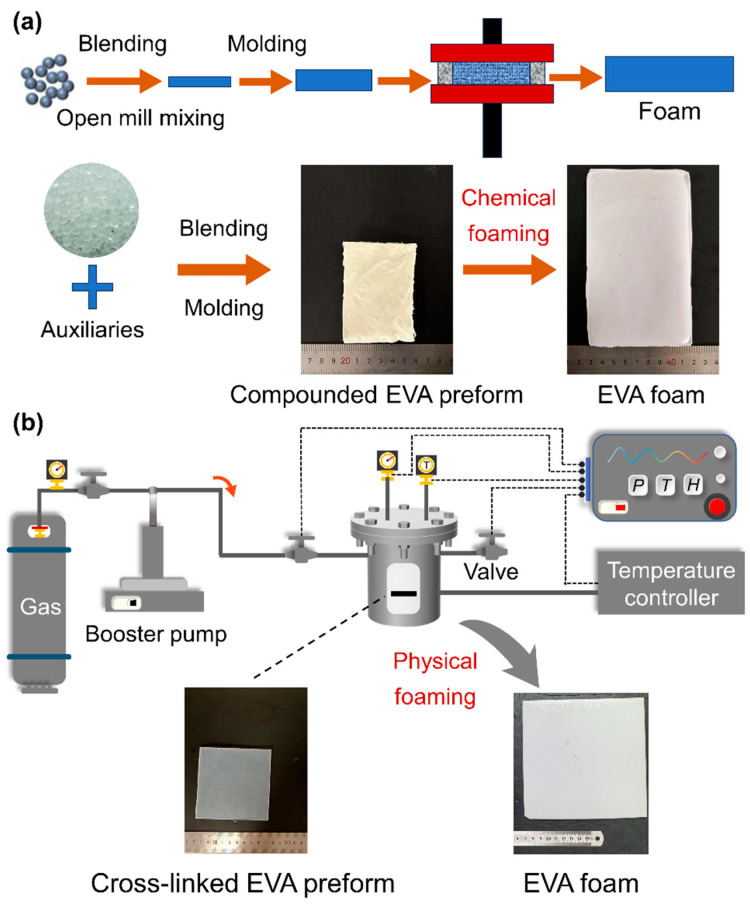
Schematic of the EVA foaming process. (**a**) Chemical foaming; (**b**) physical foaming.

As depicted in Figure 1a, EVA pellets and foaming auxiliaries were uniformly mixed in a two-roll mill at a temperature of 70 °C. At the end of the mixing process, the BIPB crosslinking agent was introduced to prevent premature crosslinking. The rolled EVA sheets were subsequently resized and compressed using a hot compression press. The stacked sheets were subjected to compression at a temperature of 110 °C and a pressure of 10 MPa for a duration of 8 min, thereby obtaining a compounded EVA preform. EVA formulation designs for the chemical and physical foaming are provided in Table 1. The samples were designated as “E-P/C-nB”, where E means EVA, P, and C denote physical and chemical foaming, respectively, and n represents the content of BIPB.

For chemical foaming, the compounded EVA preform was placed in a hot press molding machine at temperatures ranging from 165 to 190 °C under 10 MPa for 6 min to complete the foaming process. In contrast, for physical foaming, crosslinked EVA preform underwent a secondary compression process at a higher temperature of 180 °C under the same pressure for 6 min, completing the crosslinking process. N_2_ was chosen as the physical blowing agent. As illustrated in Figure 1b, EVA preform sheets were placed in a high-pressure vessel and subjected to saturation under designated conditions for 30 min to achieve full gas equilibrium. The pressure was then rapidly released within 3 s, and the foams were removed from the vessel within 3 min. These foams were stored under laboratory conditions for at least 7 days before further characterization.

### 2.3. Characterization

The vulcanization characteristics of EVA compounds were evaluated using oscillatory measurements in a moving die rheometer M-3000AU (GOTECH Testing Machine Co., Ltd., Dongguan, China).

The gel content was determined by refluxing the samples in xylene within a 400-mesh copper wire cage at 160 °C for 72 h, following ASTM D-2765 guidelines. After the extraction cycle, the residual insoluble sample was dried in a vacuum oven at 70 °C until a constant weight was achieved. The gel fraction was calculated as follows:

(1) $$Gel\;content=\frac{W_{1}-(W_{2}-W_{3})}{W_{1}}\times 100\%$$

where *W*_1_ is the original weight of the sample and *W*_2_ and *W*_3_ are the weights of the sample and the wire cage before and after extraction, respectively. The average gel content under specific crosslinking conditions was determined by analyzing three samples.

Melting and crystallization behaviors were studied using a NETZSCH DSC 204F1 differential scanning calorimeter (DSC) under a dry nitrogen atmosphere. Samples (3–5 mg) were initially heated to 180 °C and held at this temperature for 5 min to eliminate thermal history. Samples were then cooled to −50 °C. Subsequently, the samples were reheated from −50 °C to 180 °C at a heating rate of 10 °C/min. The melting temperature (*T*_m_) was identified from the endothermal peak during the second heating cycle. The crystallization temperature (*T*_c_) was taken from the exothermal peak during the cooling cycle.

Thermal gravimetric analysis (TGA) was conducted on a NETZSCH TGA 209F1 thermal gravimetric analyzer in the nitrogen atmosphere with a platinum pan. The samples were about 5 mg each and tested from 40 °C to 600 °C at a heating rate of 10 °C /min.

The crosslink density was estimated using swelling experiments, where cured EVA samples were immersed in cyclohexane at room temperature for 48 h. The Flory−Rehner equation was applied as follows:

(2) $$v_{c}=-\frac{1}{v_{s}}\frac{\ln\left(1-V_{R}\right)+V_{R}+\chi V_{R}^{2}}{V_{R}^{1/3}-2V_{R}/f}\times 100\%$$

where $v_{s}$ is the molar volume of the swelling solvent, $V_{R}$ is the volume fraction of the swollen network, χ is the polymer−solvent interaction parameter, and f represents the functionality of the network, typically assumed to be 4 [36].

The N_2_ sorption experiment was conducted in an autoclave at 120 °C. Specimens were initially weighed and placed within the autoclave, which was then flushed with N_2_ for 2 min. High-purity N_2_ was introduced to achieve a pressure of 10 MPa, 15 MPa, and 20 MPa. After saturation, the autoclave was cooled, and the specimens were removed and reweighted to determine the amount of N_2_ dissolved.

For desorption measurement, completely saturated specimens were removed from the autoclave and weighed at room temperature under atmospheric pressure at regular intervals until they reached equilibrium. The desorption diffusivities were calculated from the recorded data using the following Equation (3):

(3) $$\frac{M_{d}}{M_{\infty }}=1-\frac{4}{l}\sqrt{\frac{D_{d}\cdot t_{d}}{\pi }}$$

where *M_d_* is the amount of gas dissolved in the specimens at time *t_d_* during the desorption process. *M_∞_* is the total sorption amount of N_2_ absorbed, which can be obtained by extrapolating *M_d_* to *t_d_* = 0. *l* is the thickness of the specimens. Thus, the diffusivity of desorption *D_d_* can be estimated from the slopes of *M_d_* versus $\sqrt{t_{d}}$ plot under various pressures.

The density of the EVA foams was quantified using a densimeter (Model DA-300 M, DahoMeter, Shenzhen, China) with an accuracy of 0.005 g. The expansion ratio of the EVA foams was determined as follows:

(4) $$\phi =\frac{\rho_{s}}{\rho_{f}}$$

where *ρ*_s_ and *ρ*_f_ are the densities of the unfoamed and foamed EVA sample, respectively.

A scanning electron microscope (SEM, EM 30AX Plus, COXEM, Daejeon, Korea) was used to analyze the cell structure of the EVA foams.

## 3. Results and Discussion

### 3.1. Basic Properties of EVA

#### 3.1.1. Vulcanization Characteristic

**Figure 2:**
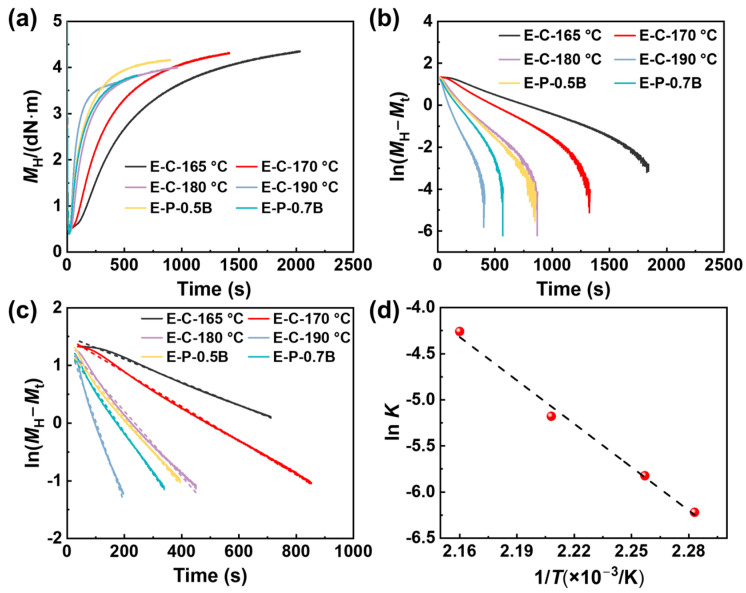
The vulcanization characteristics and the kinetics analysis of EVA at different temperatures. (**a**) Dynamic vulcanization curve of odorless BIPB at different dosage, (**b**,**c**) were the vulcanization rate of EVA-BIPB where ln (*M*_H_ − *M*_t_) varies linearly with time (**d**) analysis at specified crosslinking degrees by plotting ln K − 1/T and performing fitting.

EVA is an elastomer known for its low melting temperature and low crystallinity [1]. During the foaming process, generating a crosslinking structure is crucial to improve the foaming behavior and properties of EVA foams. The isothermal vulcanization characteristics of EVA at temperatures ranging from 165 to 190 °C were tested to identify the optimal pre-curing temperature and time. These results are presented in Figure 2 and Table 2.

The vulcanization rate (*v*) is determined by analyzing the change in dwell torque from the vulcanization curve, and the *v* is determined using:

(5) $$v=-\frac{d\left(M_{H}-M_{t}\right)}{dt}=K{\left(M_{H}-M_{t}\right)}^{n}$$

where *M*_H_ and *M*_t_ denote the maximum torque and the torque at time (*t*), respectively; *K* represents the reaction rate constant, while *n* signifies the reaction order. Assuming a first-order reaction (*n* = 1) for the vulcanization reaction of EVA-BIPB during the thermal vulcanization period, which corresponds to the crosslinking reaction stage (between the scorch time *t*_10_ and the cure time *t*_90_), the aforementioned kinetic equation can be transformed into the following Equation:

(6) $$\ln\left(M_{H}-M_{t}\right)=B-Kt$$

The formula includes *B* as the integration constant. Utilizing the data from Figure 2a to create an ln(*M*_H_ − *M*_t_) − *t* plot, as depicted in Figure 2b. The data between *t*_10_ to *t*_90_ from Figure 2c was selected for linear regression, resulting in the slope of which yielded the *K* value, as shown in Table 3. The activation energy in the initial stage of vulcanization reaction can be calculated using the logarithmic Arrhenius equation as follows:

(7) $$\ln K=lnA-\frac{E}{RT}$$

In Equation (7), *A* represents the pre-exponential factor, *R* denotes the molar gas constant with a value of 8.31 J/(mol·K), and *T* represents the absolute temperature. By plotting ln*K* − 1/*T* and performing fitting, as illustrated in Figure 2d, the reaction rate of EVA during the crosslinking reaction stage follows the Arrhenius equation. The calculated activation energy for the first-order reaction during the vulcanization crosslinking period is 93.2 kJ/mol, which is a relatively high value for the reaction. As *T* rises, the rate of vulcanization of EVA is observed to be significantly dependent on the temperature.

The *K* values were relatively small at temperatures of 165 and 170 °C, indicating a slow vulcanization reaction rate [37]. At 180 °C, the vulcanization reaction rate is moderate, thereby reducing the time required for vulcanization [38,39]. As the temperature increased up to 180 and 190 °C, the *K* values increased accordingly, suggesting an increased vulcanization rate.

**Figure 3:**
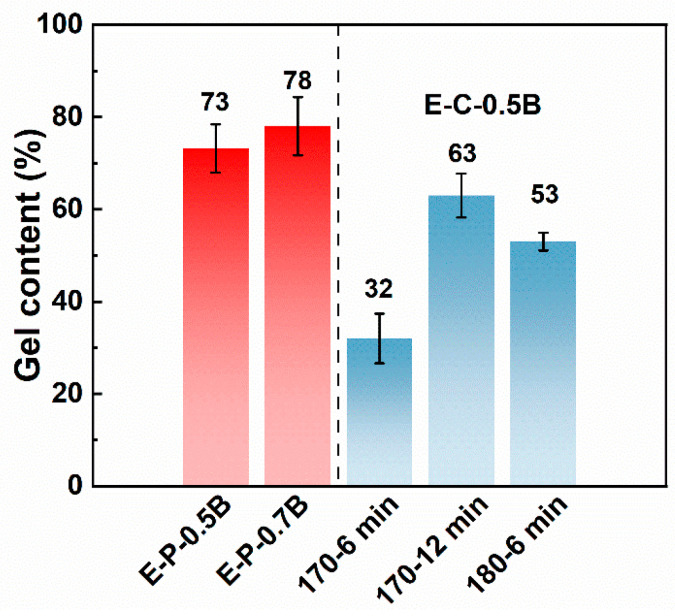
The gel content of EVA formulations. (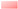) solid crosslinked EVA sheet used for physical foaming; (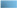) EVA foams prepared using chemical foaming.

In this work, BIPB was selected as the crosslinking agent. It is well known that the crosslink network induced using BIPB contains C−C bonds, likely resulting in higher thermal stability relative to that induced using a sulfur-cured system [40]. The crosslinking structure of EVA was adjusted by controlling the concentration of BIPB. Figure 3 shows the gel content of EVA after vulcanization and chemical foaming. E-P-0.5B resulted in a significant torque value of 3.85 dN·m (Table 2). This indicated that the molecular weight of polymer chains increased because of branching, which led to the formation of a three-dimensional crosslink network during the crosslinking process.

As the BIPB loading increased from 0.5 phr to 0.7 phr, the cured samples with a crosslink density of 66 mol/m^3^ and a gel content of 78% were generated. The presence of chain junctions led to an increase in melt viscosity, resulting in an increased torque from 3.85 to 4.50 dN·m. A further increase in BIPB loading tended to increase the gel content, crosslink density, and torque gradually. At the highest BIPB loading, the as-obtained E-P-1.2B exhibited a gel content of 91%, a crosslink density of 217 mol/m^3^, and a torque of 4.39 dN·m. These results demonstrated that the increase in BIPB loading improved the perfection of the EVA’s crosslink network. However, testing the foams obtained through chemical foaming revealed that, under the same BIPB concentration, the gel content was consistently lower than that of the sulfurized samples produced via physical foaming. This is in accordance with the vulcanization curve in Figure 2a. It can be inferred that during the chemical foaming process, along with the decomposition of the foaming agent and the chemical crosslinking process, under specific vulcanization temperature conditions, the vulcanization reaction is not fully completed when *t* is less than *t*_90_. Hence, the vulcanization efficiency does not reach its maximum value. Additionally, the uneven dispersion of additives may lead to an irregular distribution in the matrix network after vulcanization, which can affect the final test results of the vulcanization curve.

When the chemical foaming conditions were at 170 °C for 6 min, the degree of vulcanization was much lower than *t*_90_, resulting in a gel content of only 32% (Figure 3). However, when the vulcanization foaming time was 12 min, the gel content significantly increased to 63%. At a temperature of 180 °C, where the sample’s *t*_90_ was 449.6 s, the processes of crosslinking reactions and foaming agent decomposition were more synchronized, resulting in a gel content of 53% [41]. These findings suggest that there may indeed be disparities in the degree of crosslinking between samples subjected to physical foaming and those undergoing chemical foaming. During the pre-treatment process, samples subjected to physical foaming may develop a denser and more stable crosslinked network in a shorter duration, as the crosslinking reactions largely depend on external conditions like temperature or pressure and are less influenced by other variables. In contrast, samples undergoing chemical foaming might be impacted by factors such as additive dispersion, reaction rates, and uniformity, potentially leading to a less complete or uniform formation of the crosslinked network [37,42]. Therefore, theoretically, samples subjected to physical foaming might exhibit a higher degree of crosslinking.

#### 3.1.2. Thermal Properties

**Figure 4:**
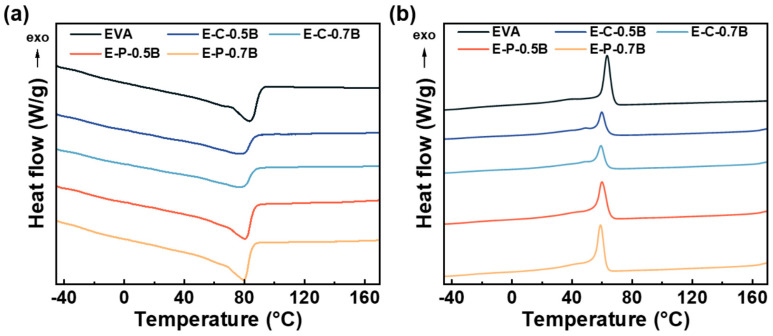
Thermal behavior of raw EVA resin and the crosslinked EVA samples. (**a**) second heating curve and (**b**) cooling curve.

Figure 4a,b display the DSC thermographs of the samples, and Table S1 details key thermal properties such as the melting point (*T*_m_), crystallization temperature (*T*_c_), and melting enthalpy (Δ*H*_m_). The analysis revealed that EVA, before and after crosslinking, shows different thermal characteristics. Initially, EVA exhibits a high melting point of 83.2 °C, a crystallization temperature of 63.3 °C, and a melting enthalpy of 74.8 J/g. Upon increasing the BIPB loading to 0.7 phr, significant changes were observed in these thermal properties: the melting point decreased to 77.2 °C, the crystallization temperature to 59.3 °C, and the melting enthalpy dropped to 41.4 J/g for sample E-C-0.7B. The reduced mobility of the polymer chains, hindered by the crosslink forming between neighboring chains, likely caused this restriction in segmental rearrangement, resulting in lower *T*_m_ and *T*_c_.

With vulcanization, crosslinked EVA samples showed a progressive decline in *T*_m_, *T*_c_, and Δ*H*_m_. This trend was similar in both physical and chemical foaming samples, although the rate of change was less pronounced in samples produced using physical foaming. The difference in the rate of thermal property alteration between the two foaming methods could be attributed to the use of inorganic additives like ZnO and CaCO_3_ in the chemical foaming samples.

Figure S1 shows the thermogram curves and the DTG curves of the EVA foams produced using a different foaming method. Table S2 indicates the weight loss, the DTG peak temperatures–*T*_p_ (maximum degradation rate), and the residual masses that were taken from Figure S1b. As seen, EVA degradation occurs in two very distinct stages. The first loss of mass is in the temperature range between 320 and 390 °C, and is related to deacetylation of vinyl acetate units [15]. The second loss of mass involves the polyethylene chains of the copolymer (ethylene C-C and C-H bonds), and occurs between 403 °C and 496 °C [43].

#### 3.1.3. Gas Solubility and Diffusivity

**Figure 5:**
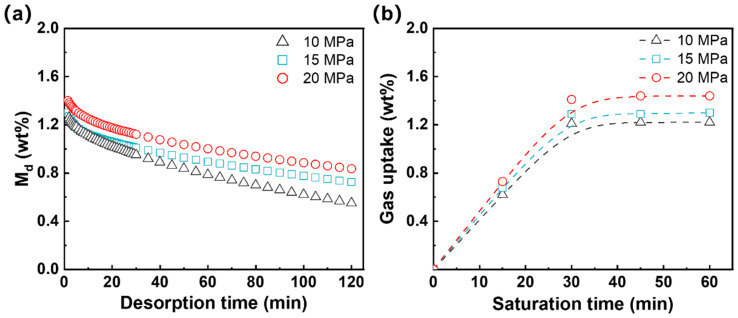
Desorption of N_2_ in E-P-0.5B after saturation for 30 min at different N_2_ pressures. The saturation temperature was 120 °C (**a**). Plots of gas concentration in E-P-0.5B as a function of saturation time at 120 °C (**b**).

N_2_ exhibits low diffusivity, which has been used with CO_2_ in mixed gasses by researchers to resist the foam shrinkage [44,45]. Figure 5 plots the mass change in the crosslinked EVA samples following saturation at varying N_2_ pressures over time. The quantitative data are listed in Table 4.

As indicated in Figure 5b, the uptake of N_2_ within the EVA specimen increases linearly with an increase in the saturation time, reaching the equilibrium solubility within the 2 mm thick sheet. Table 4 presents the N_2_ solubility in the crosslinked EVA at various pressures. At 10 MPa, the solubility is about 1.35%. It increases to 1.56% and 1.80% at 15 and 20 MPa, respectively. These results suggest that an elevated increase in saturation pressure did not result in a substantial increase in gas solubility. Table 4 also shows that the *D*_d_ of N_2_ in the crosslinked EVA is about 2.55 × 10^−12^–3.45 × 10^−12^ m^2^/s, which was about four orders of magnitude lower than the CO_2_ in elastomer [25,46]. The extremely low gas solubility and gas diffusivity of N_2_ will significantly affect the foaming behavior of the crosslinked EVA. Different from the N_2_ physical foaming, the gas content can be controlled during chemical foaming by adjusting the loading of AC content. In the following section, we will focus on the foaming behavior of EVA during the chemical process and physical processes.

### 3.2. The Foaming Behaviors of EVA during the Chemical and Physical Processes

During the chemical foaming of the EVA formulation, the generated gas during the heat-induced decomposition of AC acts as the blowing agent, and the BIPB is crosslinked in-line during the process. In contrast, during the physical foaming process, full crosslinking of EVA is required, and the dissolved N_2_ under high pressure is used as a physical foaming agent. Figure S2 shows the visual differences between the physical and chemical foaming processes. For compounded EVA preform, it appears light yellow before the foaming process because of the addition of yellow AC powers, while the samples for physical foaming exhibit a higher level of transparency. After foaming, due to the formation of cells, both types of foams become white and opaque. The chemically foamed samples, containing a small amount of TiO_2_, display a more pronounced white color.

#### 3.2.1. The Foaming Temperature Window of EVA

**Figure 6:**
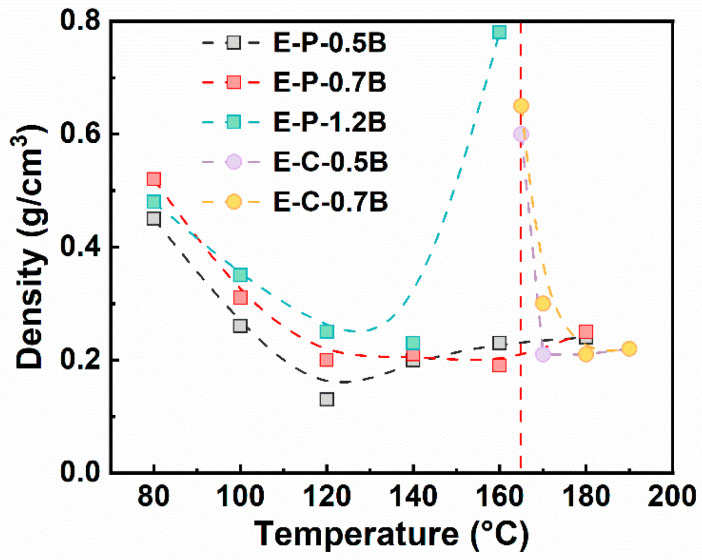
Density of EVA foams prepared using chemical and physical foaming at various foaming temperatures, where the saturation pressure was 15 MPa for the physical foaming.

Figure 6 shows the density of EVA foams fabricated using the chemical and physical foaming, where the formulations had different BIPB contents, and various temperatures were applied to obtain foams with densities lower than 0.5 g/cm^3^. EVA foams prepared using chemical foaming exhibit a narrow foaming window of about 170–190 °C, and a low temperature of 165 °C led to EVA foams with high density of 0.6–0.7 g/cm^3^, which is unacceptable for soft EVA foam. The main reason is that the low temperature of 165 °C was not high enough to induce the generation of lots of blowing agent with the thermal decomposition of AC. Meanwhile, the poor crosslinking degree of EVA formulation, as shown in Figure 3, may induce the occurrence of cell coalescence during the foaming process.

Figure 6 also shows that EVA foams produced using physical foaming present a broad foaming temperature window of about 80–180 °C, and a low density of about 0.2 g/cm^3^ can be fabricated a temperature of about 120–180 °C. Furthermore, E-P-0.5B foam has a lowest density of 0.13 g/cm^3^, which was lower that the density of EVA foams fabricated by the chemical method, i.e., 0.22 g/cm^3^, although the saturation pressure applied during the physical process was only 15 MPa. The broad processing temperature, low foaming temperature, and low N2 pressure ensure physical foaming with low energy consumption and low equipment investment. This results in low processing temperature sensitivity, constituting a significant technological advantage for the physical foaming of EVA.

CO_2_ and N_2_ are commonly used as physical blowing agents for elastomer foaming [4,47], with N_2_ typically exhibiting low gas solubility and diffusivity. Producing a low-density EVA foam at a low gas pressure of 15 MPa indicates that only a small amount of N_2_ is necessary to achieve significant expansion. This is due to the minimal escape of N_2_ and reduced post-shrinkage during foaming, attributed to N_2_’s low gas diffusivity. Another advantage of using N_2_ is that building high N_2_ pressure is relatively easier compared to CO_2_, which lowers the investment required for the gas injection system.

#### 3.2.2. Influence of Foaming Temperature on the Cell Morphology

**Figure 7:**
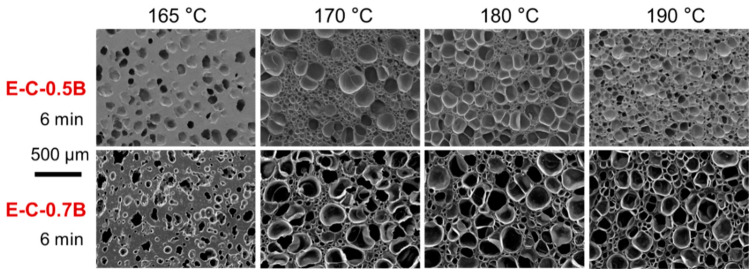
SEM images of EVA foams prepared at different foaming temperatures and BIPB loading contents where the EVA formulations were foamed using the chemical method.

**Figure 8:**
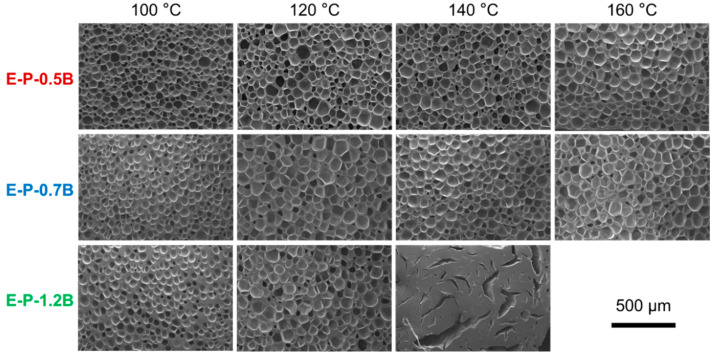
SEM images of EVA foams prepared at different foaming temperatures and BIPB loading contents, where the EVA formulations were foamed using the physical method.

Figure 6 suggests that treatment temperature is a critical parameter to control the foaming behavior of the EVA formulation. Physical foams exhibit an inverted U-shaped curve as a function of foaming temperature. The evolution of cell morphologies with temperature was further investigated, and Figure 7 shows the results of the chemical method, while Figure 8 shows the results of the physical method. Additionally, the influence of other parameters, such as the BIPB loading content, was also investigated in conjunction with the treatment temperature.

Figure 7 shows that most of EVA foams fabricated using the chemical process present a non-uniform cell structure and a dual cell size distribution. The size of large cells is about 300–400 μm, and the size of small cells is about 50–100 μm. An increase in treatment temperature does not uniformize the cell structure distribution. As the BIPB loading was increased, it appeared that the number of cells with large size increases, resulting in a deterioration of cell structure uniformity. At a foaming temperature of 165 °C and a foaming time of 6 min, it is evident that the degree of crosslinking was significantly lower compared to that at *t*_90_. Consequently, under these conditions, insufficient crosslinking of molecular chains resulted in a weak network that fails to adequately encapsulate the gasses released during the foaming agent’s decomposition. This deficiency leads to the formation of thicker cell walls, irregular cell morphology, and increased spacing between cells.

Figure 8 shows the cell morphologies of EVA foams fabricated using the physical method, where three BIPB contents were adjusted. It is evident that the EVA foams fabricated under a range of conditions and with varying BIPB loading exhibited a uniform cell structure. An increase in foaming temperature tended to result in a slight or general increase in cell size, which was associated with a change in foam density. Meanwhile, an increase in BIPB content did not dramatically alter the cell structure of EVA foams. Interestingly, the BIPB loading can reach as high as high 1.2 phr in the physically foamed EVA formulation, which was much higher than 0.7 phr used in the chemical foaming method. A higher BIPB content typically results in greater gel content and a more robust crosslinking network. This enhances the thermal stability and reduces the compression set of EVA foams, potentially improving the performance and versatility of physically foamed EVA in formulation design.

Figure 8 also shows that E-P-1.2 B foams have the density of about 0.24–0.27 g/cm^3^ at a foaming temperature of 100–140 °C. However, at a temperature of 160 °C, E-P-1.2B was no longer capable of foaming, as evidenced by the appearance of a crack-shaped structure across the sample. A similar structure has been observed in EPDM foam with high gel content and high foaming temperature or a long foaming time, where CO_2_ was used as the blowing agent [48]. A possible explanation is that a strong extension force generated during the rapid gas diffusion at a high foaming temperature may result in the disruption of the rigid crosslinking network. This, in turn, may lead to the formation of a shrunk crack structure.

#### 3.2.3. Influence of Gas Content on the Cell Morphology

**Figure 9:**
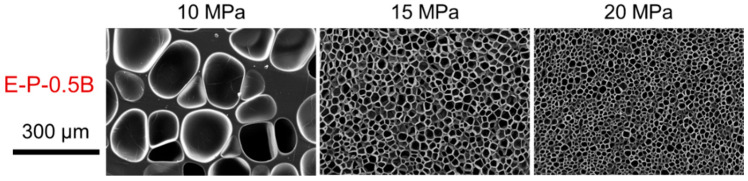
SEM images of EVA foams prepared using a physical process with the saturation temperature of 120 °C and pressures of 10–20 MPa.

Gas content is a critical parameter that affects cell morphology. According to nucleation theory, high gas content facilitates high expansion in foam production [31]. Figure 9 illustrates the cell morphology of EVA foams fabricated using the physical process, with saturation pressures ranging from 10 to 20 MPa and a saturation temperature of 120 °C. It was clearly observed that all E-P-0.5B foams exhibited a uniform cell structure. An increase in gas content, in conjunction with a rise in saturation pressure, notably increased cell density and decreased cell size. This phenomenon is very normal for the physical foaming, both in the plastic foaming system and the elastomer foaming system [31]. It is postulated that both the increased gas content and gas depressurization rate contribute to enhanced cell nucleation and increased cell density. A reduction in cell size enhances the foam’s capacity to resist deformation, which is expected to improve its mechanical properties and elasticity [44].

#### 3.2.4. Mechanisms of Cell Structure Evolution

Two review papers on the physical foaming of elastomers were recently published by our group [4,47], describing the cell growth within the crosslinking network. Unfortunately, a detailed comparative study on the movement of the crosslinking network during cell growth in chemical and physical foaming processes remains insufficient.

The process of preparing polymer foams through physical foaming typically includes four key stages: formation of a homogeneous polymer-blowing agent system, nucleation of bubbles, bubble growth, and stabilization of the foam structure. Initially, blowing agents are dissolved in the polymer to achieve a uniform polymer-gas system. Subsequently, adjusting the temperature or pressure causes the bubble nucleation within the polymer matrix. As gas evolves within the system, it diffuses into existing bubble nuclei, facilitating gradual bubble growth. When adjacent bubbles of varying sizes coalesce, gas tends to diffuse from smaller to larger bubbles due to the inverse relationship between gas pressure and bubble radius. Consequently, as bubbles expand, internal gas pressure increases, causing the bubble walls to thin and enlarging the specific surface area and volume of the polymer foam. Therefore, bubble walls must exhibit adequate strength to contain the gas and prevent structural collapse. Finally, the sample is removed and cooled to allow the polymer matrix to solidify, thereby stabilizing the size and distribution of bubbles within the foam structure [4].

**Figure 10:**
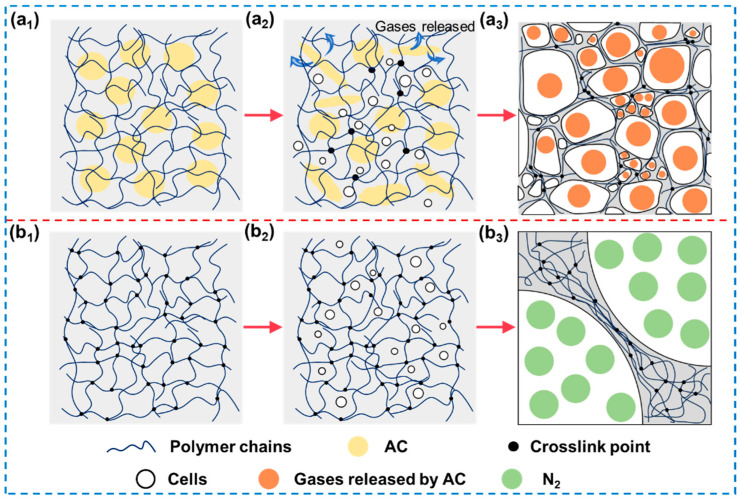
Mechanisms involved in cell formation and growth within the cross-linked networks during the chemical and physical foaming methods. The pre-foam stage in chemical (**a_1_**), the simultaneous occurrence of cross-linking reactions and foaming agent decomposition during chemical foaming (**a_2_**), and a schematic representation of the structure obtained (**a_3_**); the pre-foam stage in physical foaming (**b_1_**), the nucleation process of bubble formation during saturation (**b_2_**), a schematic diagram depicting the foam structure obtained (**b_3_**).

When the content of the crosslinking agent is equivalent, the densities of EVA foams produced via physical and chemical foaming processes were similar (Figure 6). However, significant differences were observed in cell size and distribution. Figure 10 describes the movement of the crosslinking network during the foaming processes. In chemical foaming, granular-shaped AC and BIPB with the micro-size were dispersed within the EVA formulation. Thermal treatment tended to induce gas generation and partial crosslinking. The dispersion state of AC and BIPB primarily influence where cells form and the degree of crosslinking in local chain networks. In chemical foaming, crosslinking and foaming reactions are competitive processes. If crosslinking is insufficient, gas produced by the decomposition of the foaming agent escapes rapidly due to the low strength of the EVA matrix, posing challenges in maintaining cell structure stability and achieving a stable foam material. When the degree of crosslinking is excessive, the gas produced by the decomposition of the foaming agent escapes through incompletely crosslinked gaps, hindering the cell growth.

However, during the physical foaming process, nanoscale N_2_ molecules can disperse the fully crosslinked EVA matrix (Figure 10(b_2_)). The elevated solubility of gas at high saturation pressures and adjusted temperatures did not markedly influence the behavior of gas dispersion. The rapid depressurization rate, occurring within a timeframe of approximately 0.1–0.3 s, supports uniform cell nucleation and growth, leading to a uniform cell structure [34]. Additionally, the complete pre-crosslinking of the EVA matrix promotes a uniformly crosslinked network, enabling nucleated cells to grow uniformly.

## 4. Conclusions

In this study, a comparative study of EVA foaming was carried out to investigate the differences between chemical and physical methods. EVA formulations with controlled contents of AC and BIPB were designed, and various foaming parameters were adjusted to produce EVA foams with low densities ranging from 0.15 to 0.30 g/cm^3^. The chemically foamed EVA presented a narrow foaming temperature window of about 170–190 °C, while the physically foamed EVA had a much broader foaming temperature window of about 100–180 °C. This broad temperature range facilitated EVA foaming using compressed N_2_, which reduced energy consumption and lessened the precision required for equipment temperature control. The chemically foamed EVA possessed a large cell size of about 300–400 μm and non-uniform cell size distribution resulting from the micro-scale dispersed AC and BIPB. An increased BIPB loading content tended to worsen its dispersion, leading to greater non-uniformity in cell structure. In contrast, the N_2_ molecules with nanoscale size could be well dispersed in the cross-linked EVA matrix during the pressure-quenching foaming process, inducing uniform cell nucleation and resulting in smaller cell size and uniformly distributed cell structure.

## Figures and Tables

**Table 1:** Formulation designs for the chemical and physical foaming of EVA.

	Chemical Foaming		Physical Foaming		
Samples	E-C-0.5B	E-C-0.7B	E-P-0.5B	E-P-0.7B	E-P-1.2B
EVA (phr)	100		100		
BIPB (phr)	0.5	0.7	0.5	0.7	1.2
TiO_2_ (phr)	3		0		
CaCO_3_ (phr)	15		0		
ZnO (phr)	2		0		
SA (phr)	1		0		
AC (phr)	1.35		0		

**Table 2:** Crosslink parameters of the crosslinked EVA.

Sample	*t*_90_ (s)	*M*_H_ (dN·m)	*M*_L_ (dN·m)	Crosslink Density (mol/m^3^)	Elastic Torque (dN·m)
E-P-0.5B-180 °C	394.4	4.16	0.30	45	3.85
E-P-0.7B-180 °C	338.7	4.81	0.30	66	4.50
E-P-1.2B-180 °C	303.5	4.62	0.28	217	4.39
E-C-0.5B-165 °C	1645.6	4.18	0.34	/	3.92
E-C-0.5B-170 °C	853.1	4.28	0.32	/	3.86
E-C-0.5B-180 °C	449.6	3.97	0.28	/	3.69
E-C-0.5B-190 °C	196.0	3.66	0.27	/	3.39

**Table 3:** Kinetics data of vulcanization of EVA.

Sample	*T* (°C)	*K* (s^−1^)	R-Square
E-C-0.5B	165	0.00199	0.998
	170	0.00296	0.999
	180	0.00564	0.992
	190	0.01420	0.992
E-P-0.5B	180	0.00594	0.992
E-P-0.7B	180	0.00720	0.996
E-P-1.2B	180	0.00660	0.998

**Table 4:** The solubility and desorption coefficients of N_2_ in the crosslinked EVA sheets at 120 °C.

Pressure (MPa)	Solubility (%)	D_d_ (m^2^/s)
10	1.35 ± 0.11	(2.55 ± 0.07) × 10^−12^
15	1.56 ± 0.14	(3.08 ± 0.12) × 10^−12^
20	1.80 ± 0.15	(3.45 ± 0.09) × 10^−12^

## Data Availability

Data will be made available on request.

## Supplementary Materials

The following supporting information can be downloaded at: https://www.mdpi.com/article/10.3390/ma17153719/s1, Figure S1: (a) TG curves and (b) DTG curves for the chemical foams and physical foams, Figure S2: Macroscopic images of the samples between chemical and physical foaming. (a) Chemical foaming, (b) physical foaming; Table S1: Thermal behavior of the crosslinked EVA, Table S2: TG and DTG data for the chemical foams and physical foams, Table S3: Comparison of reported foam density with the present work. References [18,43,48,49,50,51,52,53] are cited in Supplementary Materials.
