# Supplementary material for: Comparative Study of the Foaming Behavior of Ethylene–Vinyl Acetate Copolymer Foams Fabricated Using Chemical and Physical Foaming Processes
Source: Materials (Basel). 2024 Jul 27;17(15):3719. doi: 10.3390/ma17153719 (PMC11313140; doi:10.3390/ma17153719)
Supplement: Supplementary file 1 [file materials-17-03719-s001.zip › materials-3074967-supplementary.pdf]

Supporting Information

# Comparative Study of the Foaming Behavior of Ethylene–Vinyl Acetate Copolymer Foams Fabricated Using Chemical and Physical Foaming Processes

**Yaozong Li, Junjie Jiang *, Hanyi Huang, Zelin Wang, Liang Wang, Bichi Chen and Wentao Zhai ***

School of Materials Science and Engineering, Sun Yat-sen University, Guangzhou 510275, China; liyz33@mail2.sysu.edu.cn (Y.L.); huanghanyi@sysunc.com (H.H.); wangzlin7@mail2.sysu.edu.cn (Z.W.); wangliang27@mail2.sysu.edu.cn (L.W.); chenbch23@mail2.sysu.edu.cn (B.C.)

* Correspondence: jiangjj37@mail.sysu.edu.cn (J.J.); zhaiwt3@mail.sysu.edu.cn (W.Z.)

**Table. S1** Thermal behavior of the crosslinked EVA.

| Sample | $T_m$ (°C) | $T_c$ (°C) | $\Delta H_m$ (J/g) |
|---|---|---|---|
| EVA | 83.2 | 63.3 | 74.8 |
| E-C-0.5B | 78.5 | 59.7 | 41.9 |
| E-C-0.7B | 77.2 | 59.3 | 41.4 |
| E-P-0.5B | 79.9 | 60.0 | 64.9 |
| E-P-0.7B | 79.3 | 58.9 | 62.1 |

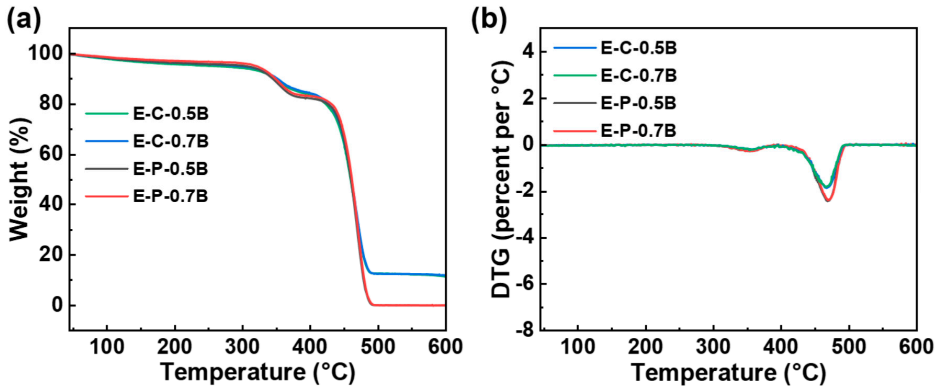


**Figure S1.** (a) TG curves and (b) DTG curves for the chemical foams and physical foams.

**Table. S2** TG and DTG data for the chemical foams and physical foams.

| Sample | [a]Tp (°C) | | Weight loss (%) | |
|---|---|---|---|---|
| | First loss | Second loss | First loss | Second loss |
| E-C-0.5B | 357.75 | 466.37 | 11.33 | 58.25 |
| E-C-0.7B | 358.40 | 467.76 | 10.96 | 62.06 |
| E-P-0.5B | 359.19 | 472.07 | 13.87 | 70.05 |
| E-P-0.7B | 355.65 | 473.46 | 14.52 | 71.12 |

[a]Tp: DTG peak temperature (maximum degradation rate).

**Figure. S1** shows the thermogram curves (a) and the DTG curves (b) of the EVA foam produced using different foaming methods. **Table S2** indicates the weight loss, the DTG peak temperatures, Tp (maximum degradation rate), and the residual mass that were taken from

**Figure. S1b**. It can be seen that EVA degradation occurs in two very distinct stages. The first loss of mass is in the temperature range between 320 and 390 °C, and is related to deacetylation of vinyl acetate units [1]. The second loss of mass involves the polyethylene chains of the copolymer (ethylene C-C and C-H bonds), and occurs between 403 °C and 496 °C [2]. The mass losses for the E-P-0.5B and of the E-P-0.7B composites in both events varied little, with no variation in the type of EVA used. Consequently, the vinyl acetate content was unchanged. However, the percentage of residual mass at 600°C increased with the addition of processing aid to the EVA.

For the second degradation phase, the DTG peak temperature (Tp) of E-C and E-P composite materials remains nearly unchanged, indicating that additives do not influence the degradation process of polyethylene chains within the copolymer. The second stage of degradation involves the complete rupture of residual main chains, depending on the relationship between the crosslinking and EVA units. The degradation mechanism of EVA correlates directly with the percentage of vinyl acetate, yet its behavior during degradation relates to higher DTG peak temperatures and residual masses. This is evidently due to the inclusion of high-temperature-resistant additives such as ZnO and $CaCO_3$, as evidenced by the remnants remaining at 600 °C.

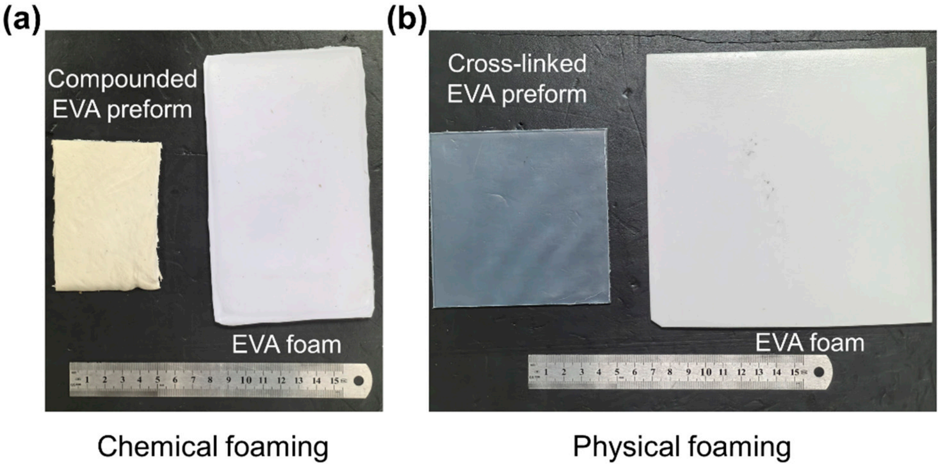


**Figure S2.** Macroscopic images of the samples between chemical and physical foaming. (a) Chemical foaming, (b) physical foaming.

EVA foams typically exhibit a density range of 0.1 to 0.2 g/cm$^3$, which is notably low compared to several other materials commonly used in similar applications. Specifically, when compared to materials like traditional rubber or EVA/POE chemical foams, which often have densities ranging from 0.16 to 0.25 g/cm$^3$ [3], EVA foams stand out for their lower density characteristics.

**Table S3** is a comparative list of the densities observed in our current work versus the reported densities of foams from various materials with high crosslinking content. Moreover, in applications where lightweight materials with good cushioning properties are required, such as in footwear, packaging, and sporting goods, EVA foams' low density provides distinct advantages over heavier materials like solid rubber or higher-density foams.

**Table S3.** Comparison of reported foam density with the present work.

| Sample | Density (g/cm$^3$) | Reference |
|---|---|---|
| EVA—chemical foam | 0.20–0.30 | This work |
| EVA—physical foam | 0.15–0.25 | This work |
| HD-TPU | 0.30–0.90 | [4] |
| EVA-BATTE-V foam | 0.25 | [5] |
| EVA/POE | 0.16 | [1] |
| EPDM | 0.30–0.90 | [6] |
| OBC | 0.15–0.70 | [7] |
| SEBS | 0.25 | [8] |

## References


1. Shafeeq, V. H.; Unnikrishnan, G., Experimental and theoretical evaluation of mechanical, thermal and morphological features of EVA-millable polyurethane blends. *Journal of Polymer Research* **2020,** 27 (3).
2. Hoang, T.; Chinh, N. T.; Trang, N. T. T.; Hang, T. T. X.; Thanh, D. T. M.; Hung, D. V.; Ha, C.-S.; Aufray, M., Effects of maleic anhydride grafted ethylene/vinyl acetate copolymer (EVA) on the properties of EVA/silica nanocomposites. *Macromolecular Research* **2013,** 21 (11), 1210-1217.
3. Chang, B. P.; Kurkin, A.; Kashcheev, A.; Leong, K. F.; Tok, A. l. Y.; Lipik, V., Enhancing dynamic impact performance and cushioning of EVA copolymer

foams with thermoplastic elastomers. *Materials Today Communications* **2024,** 38.

4. Chen, B.; Jiang, J.; Li, Y.; Zhou, M.; Wang, Z.; Wang, L.; Zhai, W., Supercritical Fluid Microcellular Foaming of High-Hardness TPU via a Pressure-Quenching Process: Restricted Foam Expansion Controlled by Matrix Modulus and Thermal Degradation. *Molecules* **2022,** 27 (24).
5. Cheng, L.; Liu, S.; Yu, W., Recyclable ethylene-vinyl acetate copolymer vitrimer foams. *Polymer* **2021,** 222.
6. Zheng, H.; Pan, G.; Huang, P.; Xu, D.; Zhai, W., Fundamental Influences of Crosslinking Structure on the Cell Morphology, Creep Property, Thermal Property, and Recycling Behavior of Microcellular EPDM Foams Blown with Compressed CO2. *Industrial & Engineering Chemistry Research* **2020,** 59 (4), 1534-1548.
7. Zheng, H.; Huang, P.; Lee, P. C.; Chang, N. R. S.; Zhao, Y.; Su, Y.; Wu, F.; Liu, X.; Zheng, W., Lightweight olefin block copolymers foams with shrinkable and recoverable performance via supercritical CO2 foaming. *The Journal of Supercritical Fluids* **2023,** 200.
8. Zhang, Z. X.; Dai, X. R.; Zou, L.; Wen, S. B.; Sinha, T. K.; Li, H., A Developed, Eco-Friendly, and Flexible Thermoplastic Elastomeric Foam from SEBS for Footwear Application. *Express Polym. Lett.* **2019,** 13 (11), 948-958.
